# Hepatitis E Virus Outbreak in Monkey Facility, Japan

**DOI:** 10.3201/eid1812.120884

**Published:** 2012-12

**Authors:** Hiroshi Yamamoto, Juri Suzuki, Atsushi Matsuda, Takafumi Ishida, Yasushi Ami, Yuriko Suzaki, Isao Adachi, Takaji Wakita, Naokazu Takeda, Tian-Cheng Li

**Affiliations:** Author affiliations: University of Toyama, Toyama, Japan (H. Yamamoto, I. Adachi);; Kyoto University, Aichi, Japan (J. Suzuki); University of Tokyo, Tokyo, Japan (A. Matsuda, T. Ishida);; National Institute of Infectious Diseases, Tokyo (Y. Ami, Y. Suzaki, T. Wakita, T.-C. Li);; Osaka University, Osaka, Japan (N. Takeda)

**Keywords:** Hepatitis E, outbreak, hepatitis E virus, HEV, viruses, antibodies, genotype G3, monkeys, monkey facility, zoonosis, Japan

## Abstract

An outbreak of hepatitis E virus occurred in an outdoor monkey breeding facility in Japan during 2004–2006. Phylogenetic analysis indicated that this virus was genotype 3. This virus was experimentally transmitted to a cynomolgus monkey. Precautions should be taken by facility personnel who work with monkeys to prevent infection.

Wild or reared monkeys have been used as disease models in animal facilities worldwide. Because disease caused by hepatitis E virus (HEV) is a zoonosis (*1*–*4*), monkeys might be infected. We examined the prevalence of antibodies against HEV in serum and fecal samples collected from monkeys in animal facilities at the Primate Research Institute of Kyoto University in Japan for 6 years (2004–2009). We found that spontaneous infection and transmission of HEV occurred in a monkey facility.

## The Study

There are 9 monkey colonies (A–I) at the Primate Research Institute of Kyoto University. Colonies A–G contained Japanese monkeys (*Macaca fuscata*), and colonies H and I contained rhesus monkeys (*Macaca mulatta*). Each colony was bred in a separate outdoor breeding facility. A total of 588 monkey serum samples were collected during September–November 2004–2009 and tested for IgG and IgM against HEV and for HEV RNA by ELISA or reverse transcription PCR (RT-PCR) as described (*5*–*7*). Samples from colonies G and F were collected during 2004–2006, whereas in 2009 samples were collected from colonies A, C, D, and I.

The prevalence of IgG against HEV was 0% in 2004, 20.0% in 2005, and 78.5% in 2006, followed by a gradual decrease to 35.9% in 2009 (Table 1). The prevalence of IgM against HEV increased from 0% in 2004 to 2.5% in 2005 and to 6.6% in 2006, and then decreased to 1.1% in 2007 and 0% in 2008 and 2009.

**Table 1:** Prevalence of IgG and IgM against hepatitis E virus in monkeys at monkey facility, Japan, 2004–2009

Year	No. positive/no. tested (%)	
	IgG	IgM
2004	0/110	0/110
2005	24/120 (20.0)	3/120 (2.5)
2006	96/121 (78.5)	8/121 (6.6)
2007	73/96 (76.0)	1/96 (1.1)
2008	47/90 (52.2)	0/90
2009	18/51 (35.3)	0/51

**Figure 1:**
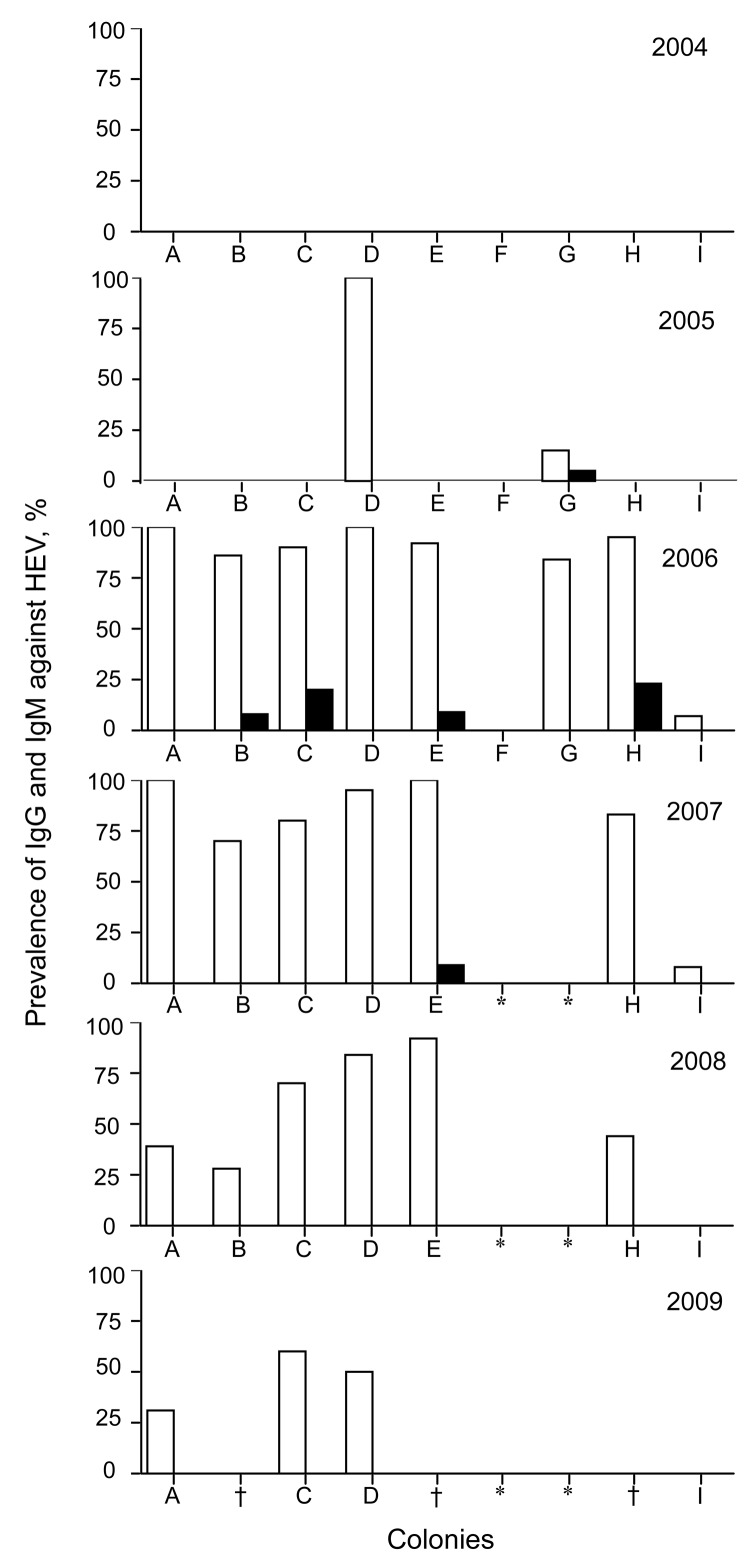
Prevalence of IgG (white bars) and IgM (black bars) against hepatitis E virus (HEV) in monkey facility, Japan, 2004–2009. *Monkeys were moved to another animal facility; †specimen not available.

IgG against HEV was not detected in any of the 9 colonies in 2004, indicating that HEV infection did not occur before October 2004. However, in 2005, the prevalence of IgG reached 100% in colony D and 20% in colony G (Figure 1). ELISA titers were high, ranging from 0.293 to 1.641 in colony D and from 0.230 to 0.845 in colony G. These results suggested that HEV infection occurred after October 2004 in the monkey facility. The prevalence of IgG was higher in colony D than in colony G, and IgM was not detected in colony D, suggesting that HEV infection occurred earlier in colony D than in colony G. These colonies adjoined each other, indicating that the first HEV infection occurred in colony D and was then transmitted to colony G. Colonies A, C, D, E, and H each had an IgG prevalence of 90%–100%, and colonies B and G had an IgG prevalence >80% in 2006 (Figure 1). These results demonstrated that infection spread over a large area, except for colony F, during 2005 and 2006.

To compare the kinetics of IgG formation during 2004–2009, serum samples from 25 monkeys whose peak ELISA optical density (OD) values for IgG against HEV were each higher than 1.0 were selected. In most monkeys, OD values for IgG increased rapidly and then decreased gradually year by year. The kinetic pattern of monkey M1543 was different from those of other monkeys that had high OD values (2.568–2.738). IgM was detected exclusively in this monkey in 2006 (OD value 0.620).

Serum samples from the 25 monkeys were used to detect HEV RNA by RT-PCR. Four serum samples were positive for HEV RNA; all were from the same monkey (M1543) from which samples were collected in 2006, 2007, 2008, and 2009. Nucleotide sequences of 348 bp coding the partial open reading frame 2 showed 100% identity. This result indicated that monkey M1543 was infected persistently with HEV and produced virus continuously.

To examine whether HEV was present in feces, 2 fecal samples were collected from monkey M1543 in September and November 2009 for detection of HEV RNA. Both samples were positive for HEV RNA. Nucleotide sequences of these samples were identical to those detected from serum samples.

**Figure 2:**
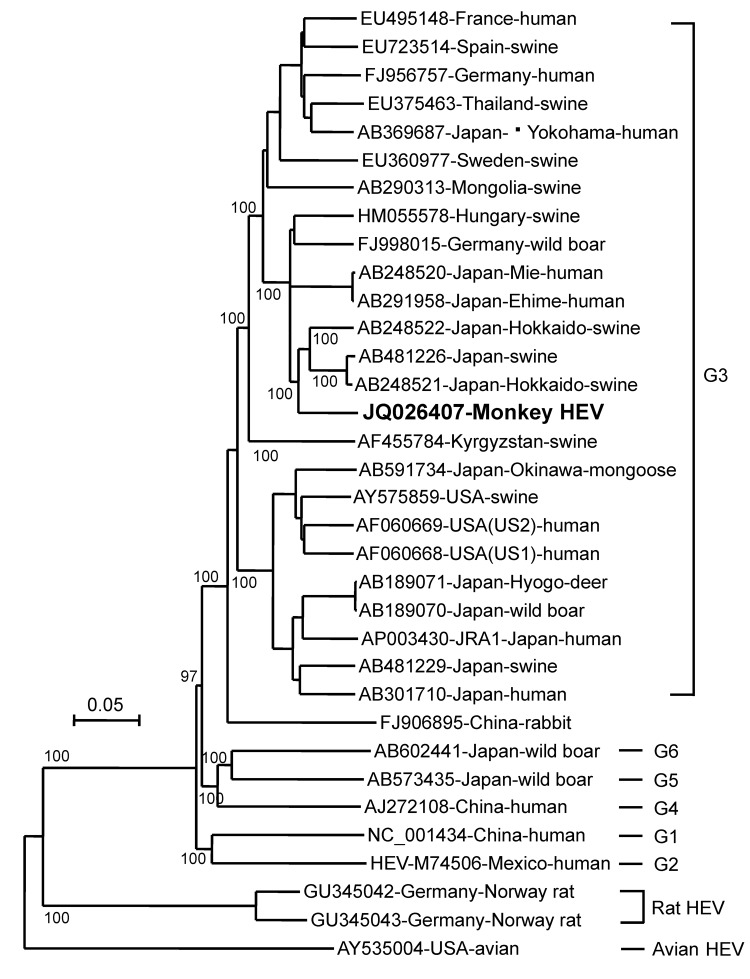
Phylogenetic analysis of monkey hepatitis E virus (HEV) Inuyama strain on the basis of nucleotide sequence of the HEV genome except for a 5′ noncoding region (7,206 nt) by using avian HEV as an outgroup. Values along the branches are bootstrap values determined on the basis of 1,000 resamplings of datasets. **Boldface** indicates strain isolated in this study. Genotypes are indicated on the right. Scale bar indicates nucleotide substitutions per site.

Primers were designed on the basis of sequences of swine HEV (GenBank accession no. AB248522), and RT-PCR was performed to amplify the viral genome except for the N terminus noncoding region. This strain was designated the monkey HEV Inuyama strain (JQ026407). Phylogenetic analysis of its genome indicated that this strain belongs to HEV genotype 3 (Figure 2). Infectivity of the monkey HEV strain was examined ex vivo with a human hepatocarcinoma cell line (PLC/PRF/5), and in vivo with 2 HEV-negative cynomolgus monkeys. Both experiments showed that the virus was infectious (Technical Appendix Figures 1 and 2).

A total of 94 human serum samples were collected from staff of the Primate Research Institute and subjected to ELISA for detection of IgG and IgM against HEV. All serum samples were negative for IgM against HEV, but the prevalence of IgG was 6.9% in 2007, 9.7% in 2008, and 11.8% in 2009, although differences among these years were not significant (p>0.05) (Table 2). No HEV RNA was detected in serum samples, and none of the staff had symptomatic hepatitis E during the 6-year study.

**Table 2:** Prevalence of IgM against hepatitis E virus in serum samples from animal handlers at monkey facility, Japan, 2007–2009*

Year	No. positive/no. tested (%)
2007	2/29 (6.9)
2008	3/31 (9.7)
2009	4/34 (11.8)

*All samples were negative for IgG against hepatitis E virus and for virus RNA.

## Conclusions

We conducted long-term monitoring of HEV infection in monkeys and report natural infection and transmission of HEV in a monkey facility. We sought to determine the source of the HEV outbreak and where HEV was introduced to colony D. At our research institute, each monkey colony is bred in a separate outdoor breeding facility built on a mountain, and the monkeys live in an environment similar to their natural habitat. Because each outdoor feeding facility is isolated by a double fence, natural reservoirs of HEV (wild boars and deer) cannot enter it. Phylogenetic analysis of monkey HEV strains indicated that this virus was genotype 3, and BLAST analysis showed that the monkey isolate is closest to HEV strains isolated from pigs in Japan. Nucleotide identities were 92%–93% (AB248521, AB248522, and AB481226). However, no evidence indicates that HEV is transmitted from pigs or wild boars to monkeys.

A notable finding in this study was the persistence of HEV infection. Generally, HEV infection is self-limiting and symptoms are transient. Persistent HEV infection occurs in solid-organ transplant recipients who have received immunosuppressive drugs (*8*) or in patients with other conditions associated with immunosuppression, such as HIV infection (*9*) and hematologic malignancies (*10*,*11*). However, there is no evidence of immunosuppression in monkey M1543, and the cause of the persistent HEV infection in this monkey is unknown.

The fact that the infectious HEV strain was detected in a monkey facility and caused an HEV outbreak cast doubt and apprehension on the safety of handling monkeys. Although no staff member showed development of symptomatic hepatitis E, precautions should be taken by facility workers who work with monkeys to prevent infection with HEV.

Technical Appendix Experimental infection of 2 cynomolgus monkeys with hepatitis E virus, Japan.
