# Supplementary material for: Hepatitis E Virus Outbreak in Monkey Facility, Japan
Source: Emerg Infect Dis. 2012 Dec;18(12):2032–4. doi: 10.3201/eid1812.120884 (PMC3557886; doi:10.3201/eid1812.120884)
Supplement: Technical Appendix — Experimental infection of 2 cynomolgus monkeys with hepatitis E virus, Japan. [file 12-0884-Techapp-s1.pdf]

Article DOI: http://dx.doi.org/10.3201/eid1812.120884

Hepatitis E Virus Outbreak in Monkey Facility, Japan

Technical Appendix

Experimental Infection of 2 Cynomolgus Monkeys with Hepatitis E Virus, Japan

Growth of Monkey Hepatitis E Virus on PLC/PRF/5 Cells

A hepatocarcinoma cell line (PLC/PRF/5) was cultured in a 25-cm^2 culture bottle containing 5 mL of Dulbecco modified Eagle medium and 10% fetal calf serum. A 10% suspension of a monkey fecal specimen (2 mL) was placed on PLC/PRF/5 cells. After adsorption at 37°C for 1 h, cells were washed 3× with phosphate-buffered saline and 8 mL of maintenance medium (medium 199; Invitrogen, Carlsbad, CA, USA) containing 2% (vol/vol) heat-inactivated fetal calf serum and 10 mmol/L $MgCl_2$. Culture medium was replaced every 3 days and used for detection of hepatitis E virus (HEV) antigen and HEV RNA.

Infectivity of Monkey HEV

Two cynomolgus monkeys (*Macaca fascicularis*), M3471 (21-year-old male) and M4530 (13-year-old male), were inoculated intravenously with 2 mL of suspension (titers of HEV RNA were unknown). Fecal samples were collected daily and used for detection of HEV RNA. Serum samples were collected weekly to detect HEV RNA, HEV-specific antibodies, and levels of alanine aminotransferase and aspartate aminotransferase. All monkey experiments were reviewed and conducted according to the Guides for Animal Experiments performed at the National Institute of Infectious Diseases (code 510001). Primates were housed individually in biosafety level 2 facilities.

Cell Culture of Monkey HEV

To examine infectivity of the monkey HEV Inuyama strain in fecal samples, a fecal suspension from monkey M1543 was placed on PLC/PRF/5 cells. HEV antigen and RNA in cell culture supernatant were detected by ELISA and reverse transcription PCR, respectively. HEV antigen was detected on day 68, and the optical density value increased exponentially until

peaking on day 110 postinfection (Technical Appendix Figure 1). HEV RNA was detected on day 50 postinfection, and the nucleotide sequence of 348-bp PCR product showed 100% identity with that of the original fecal specimen. Extensive virus growth was found constantly in the second passage, as indicated by earlier detection of HEV antigen and RNA in the culture medium (Technical Appendix Figure 1). These results indicated that monkey M1543 secreted infectious HEV.

Transmission of Monkey HEV from Japanese Monkeys to Cynomolgus Monkeys

Infectivity of the monkey HEV Inuyama strain was also confirmed by an animal experiment. Two cynomolgus monkeys (M3471 and M4530) were inoculated with 2 mL of fecal suspension, and biochemical, serologic, and virologic markers were monitored (Technical Appendix Figure 2). HEV RNA was detected within 1 week after infection in serum samples from both monkeys. HEV RNA was detected in feces on days 7–12 postinfection in monkey M3471 and on days 8–11 postinfection in monkey M4530. Increases in IgG and IgM titers were found in these animals, indicating that the HEV strain isolated from the Japanese monkey was infectious. However, neither cynomolgus monkeys showed increased alanine aminotransferase and aspartate aminotransferase levels during the experiment.

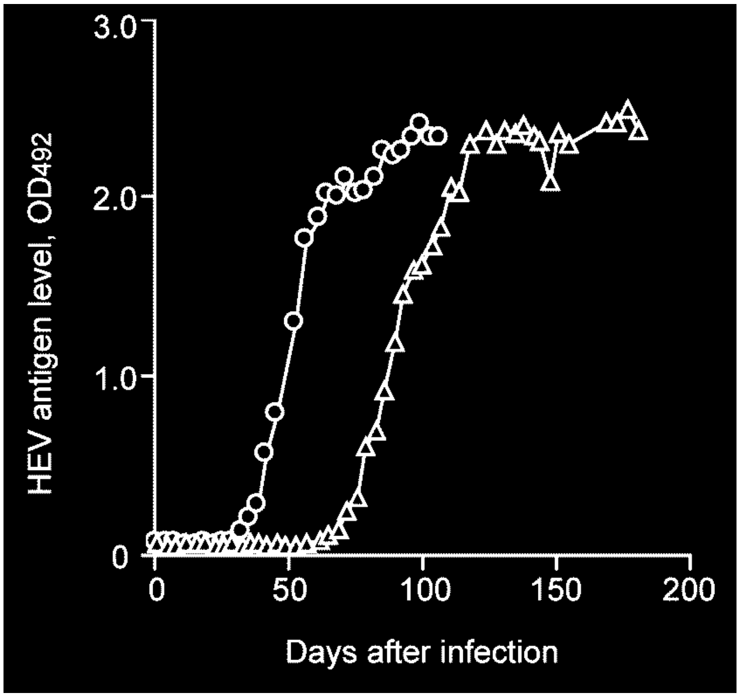

Technical Appendix Figure 1. Replication of monkey hepatitis E virus (HEV) in a hepatocarcinoma cell line, Japan. PLC/PRF/5 cells were infected with a monkey fecal specimen positive for HEV RNA or culture supernatant. Culture supernatants were collected every 3 days and used for detection of HEV antigen by ELISA. Circles indicate fecal specimen–infected PLC/PRF/5 cells, and triangles indicate culture supernatant–infected PLC/PRF/5 cells. Supernatant was collected on day 120 from fecal specimen–infected PLC/PRF/5 cells.

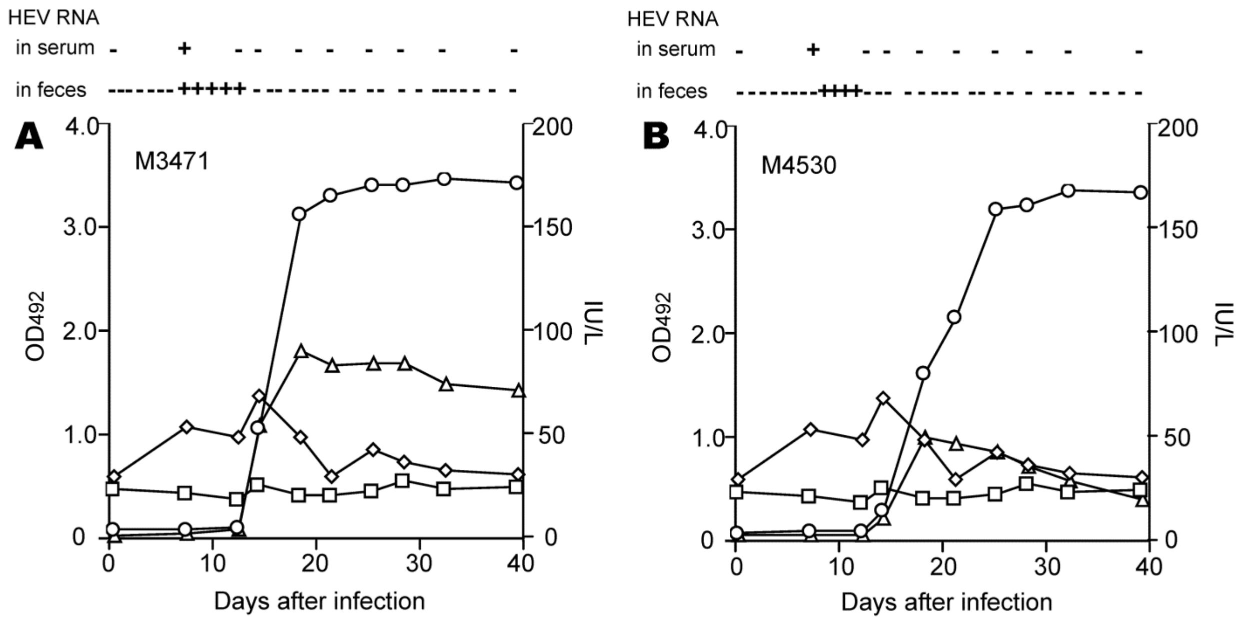

Technical Appendix Figure 2. Kinetics of biochemical, serologic, and virologic markers after challenge of 2 cynomolgus monkeys with hepatitis E virus (HEV), Japan. Monkeys M3471 (A) and M4530 (B), which were negative for HEV antibody, were infected with monkey HEV. HEV RNA in serum and fecal samples was monitored by reverse transcription PCR. Circles indicate IgG in serum, triangles indicate IgM in serum, squares indicate alanine aminotransferase levels, and diamonds indicate aspartate aminotransferase levels. -, negative: +, positive; OD_{492}, optical density at 492 nm.
